# Passive microwave Arctic sea ice melt onset dates from the advanced horizontal range algorithm 1979–2022

**DOI:** 10.1038/s41597-023-02760-5

**Published:** 2023-12-01

**Authors:** Angela C. Bliss

**Affiliations:** NASA Goddard Space Flight Center, Cryospheric Sciences Laboratory, Greenbelt, Maryland 20771 USA

**Keywords:** Cryospheric science, Ocean sciences

## Abstract

The onset of the summer melt season is a key stage of the Arctic sea ice seasonal cycle and is an indicator of climate change. Surface melting of the bare or snow-covered sea ice is detected using passive microwave satellite observations. The data set presented here is a 44 year record of Arctic sea ice annual melt onset (MO) dates for 1979–2022 produced using an updated version of the Advanced Horizontal Range Algorithm (AHRA). This data product contains annual maps of the sea ice MO date and a set of descriptive statistics summarizing the data. This paper describes a new update of the AHRA methodology, now AHRA V5, including key changes to the algorithm starting date and sea ice mask methodology to improve estimates of early-season MO dates especially near the sea ice periphery. AHRA V5 data are suitable for monitoring trends in Arctic and regional sea ice MO dates and for process studies of atmosphere-sea ice interactions during the early spring and summer months.

## Background & Summary

The date of snow melt onset (MO) on Arctic sea ice is the first day of the year that liquid water forms on the sea ice surface or in the snow pack atop the ice due to melting in the spring or early summer, marking the beginning of the summer sea ice melt season^1^. This MO date is also known as the early MO date^2,3^ which can be followed by a secondary date known as the continuous MO date after which melting of the snow and ice surface continues until autumn freeze-up. The annual date of MO on Arctic sea ice marks a key stage of the sea ice seasonal cycle and, when tracked over time, is used as an indicator of Arctic climate change^4^. Sea ice MO can be initiated by enhanced longwave radiation from thickened cloud cover^5,6^, passing cyclones^7^, or atmospheric heat^8^ and moisture^9,10^ transport and the seasonal increase of local temperatures in the air, snow, and sea ice to the melting point. Since 1979, trends in MO dates have shown that the summer melt season is occurring earlier in the year as a consequence of amplified warming in the Arctic^11^ and contributing to lengthening of the summer melt season^3^. When the MO date and associated surface albedo reductions from melting snow occur earlier in year, the total amount of insolation absorbed by the ice-ocean system throughout the summer season increases^12^, which in turn increases ocean heat content^13^ delaying the onset of sea ice freeze-up in the autumn^3^.

Many methodologies have been developed to estimate Arctic sea ice MO dates using active^14–16^ and passive microwave satellite observations^1,17–20^ as well as air temperature-based methods from satellite^11^ and *in situ*^21–23^ observations. However, passive microwave methods are often preferred to monitor sea ice properties because the observations are available Arctic-wide on a near-daily basis since late 1978 and daily since 1987 and are not limited by cloud cover or the availability of sunlight. Thus, a consistent 44+ year record of MO dates can be derived from passive microwave observations which is ideal for long-term climate change monitoring at both pan-Arctic^4^ and regional^24^ scales. Additionally, data products providing Arctic sea ice MO dates are useful for investigations of inter-annual variability of the Arctic sea ice seasonal cycle^4,24^ and for process studies of sea ice-atmosphere interactions^7,10^. Arctic MO dates are also used to flag reduced data quality in sea ice concentration (SIC) products during the melt season^25,26^ and have been used to evaluate biases in modeled Arctic sea ice in global circulation models^27,28^.

In this work, an established Arctic MO date algorithm known as the Advanced Horizontal Range Algorithm (AHRA) originally developed by Drobot and Anderson^1^ was updated to produce a new data set of Arctic sea ice MO dates, hereafter called AHRA version 5 (V5). The AHRA V5 data set benefits from a major update to the methodology which includes the introduction of variable algorithm starting dates and a new method for deriving the annual sea ice extent (SIE) mask. Modifying the algorithm starting date improves a key limitation known to exist in prior versions of the AHRA MO data product as well as in other passive microwave MO date products^29^. Key differences in the methodologies between AHRA V5 and the prior version of the AHRA data product, version 4^11,30^ (V4), are summarized in Table 1. The new methodology presented herein produces some differences in the resulting MO dates; thus, a comparison of the annual mean MO dates and examples of regional differences is presented for users already familiar with prior data versions. The AHRA V5 data product is available at the NASA Distributed Active Archive Center (DAAC) at the National Snow and Ice Data Center (NSIDC)^31^. The NSIDC archive of the AHRA V5 data set will be routinely updated with new annual MO date data in the future. Thus, a permanent archive of the AHRA V5 MO dates for 1979–2022 presented herein is available for download at Zenodo^32^. Earlier versions of the AHRA MO dates data set can be obtained by contacting NSIDC’s User Services. Data set documentation and metadata is presently available on the NSIDC website for AHRA version 3^33^ and V4^30^ and the metadata is presently available for AHRA versions 1^34^ and 2^35^.

**Table 1:** Summary of differences between AHRA V5 and V4 processing.

	AHRA V5	AHRA V4
Time Coverage	1979–2022	1979–2017
SIE Mask Definition	Annual max. SIE based on a 50% SIC threshold (SIC date range: 15 February–31 March)	50% SIC threshold on one or both of the first two days with valid SICs in March (SIC date range: 1–4 March)
Algorithm Starting Date	The day following the date of max. SIE based on a 50% SIC threshold	DOY 61
Sensor for 2017 Tbs	DMSP F18	DMSP F17

## Methods

### Passive microwave brightness temperatures and inter-sensor calibration

Passive microwave brightness temperatures (Tbs) from the Scanning Multichannel Microwave Radiometer (SMMR) onboard NASA’s Nimbus-7 platform and the Special Sensor Microwave/Imager (SSM/I) and Special Sensor Microwave Imager and Sounder (SSMIS) instruments onboard the Defense Meteorological Satellite Program (DMSP) series of F08, F11, F13, F17, and F18 platforms were used to derive the AHRA V5 MO dates. The gridded Tbs were obtained from the Nimbus-7 SMMR Polar Gridded Radiances and Sea Ice Concentrations, version 1^36^ and the DMSP SSM/I-SSMIS Daily Polar Gridded Brightness Temperatures, version 6^25^ data sets distributed by the NASA DAAC at NSIDC. The data are gridded to NSIDC’s north polar stereographic grid (304 by 448 grid cells) with a nominal spatial resolution of 25 km (https://nsidc.org/data/user-resources/help-center/guide-nsidcs-polar-stereographic-projection). The AHRA uses horizontally polarized Tbs from the 19 GHz (18 GHz for SMMR) and 37 GHz channels, hereafter noted as 19H and 37H. The temporal coverage of Tb data obtained from each satellite platform in the data set is listed in Table 2. Tbs from SMMR were collected every second day due to power limitations of the instrument and collected daily from the SSM/I and SSMIS sensors. In all cases, Tbs from a single sensor were used to derive MO dates for the year using observations from day of year (DOY) 1 through DOY 245 at the end of August which is assumed to be the last possible date for MO. The exception is 1987 when the last date of data from the SMMR sensor was collected 20 August, 11 days prior to the end of the month (Table 2).

**Table 2:** Temporal coverage of passive microwave sensors used for computation of AHRA V5 MO dates.

Platform and Sensor	Start Date	End Date
NASA Nimbus-7 SMMR	1 January 1979	20 August 1987
DMSP F08 SSM/I	1 January 1988	18 December 1991
DMSP F11 SSM/I	1 January 1992	31 December 1995
DMSP F13 SSM/I	1 January 1996	31 December 2007
DMSP F17 SSMIS	1 January 2008	31 December 2016
DMSP F18 SSMIS	1 January 2017	31 December 2022

Prior to deriving MO dates, Tbs from the different sensors are inter-calibrated to one reference sensor to create a consistent data set suitable to produce multi-decadal time series. The inter-sensor calibration was performed on the 19H and 37H Tbs using correlation coefficients (slope and intercept) derived from linear regression analyses performed during sensor overlap periods from overlap areas representative of Arctic sea ice where possible as listed in Table 3. Following Abdalati *et al*.^37^, the SSM/I on F08 is used as the reference sensor and Tbs from the other sensors are corrected to match this baseline by applying the correction equations in Table 3, in series, as needed. For example, Tbs from F17 were corrected to the F08 baseline in steps using the equations to correct F17 to F13, then F13 to F11, then F11 to F08. An analysis of Tbs from the F18 sensor found that they are highly correlated (>0.99) with Tbs from the F17 sensor with only small variations due to slight differences in the sensor crossing times^38^. Differences that do occur between F17 and F18 Tbs were found to mainly affect the very low ice concentration areas along the sea ice edge^38^; thus, no additional inter-sensor correction was performed to correct F18 Tbs to F17 for deriving the AHRA V5 MO dates, i.e., Tbs from F18 and F17 were corrected to the F08 reference using identical procedures (Table 3).

**Table 3:** Linear regression coefficients (slope and intercept) and correction equations used for inter-sensor calibration of Tbs with DMSP F08 as the baseline sensor.

Sensor correction	Source	Overlap area	Channel	Slope	Intercept (K)	Correction equation
SMMR to F08	Jezek *et al*.^50^	—	18H	0.94	2.62	$F08=\frac{SMMR-2.62}{0.94}$
			37H	0.954	2.85	$F08=\frac{SMMR-2.85}{0.954}$
F11 to F08	Abdalati *et al*.^37^	Greenland	19H	1.013	−1.89	$F08=1.013xF11-1.89$
			37H	1.024	−4.22	$F08=1.024xF11-4.22$
F13 to F11	Stroeve *et al*.^51^	NH Sea Ice	19H	0.986	2.179	$F11=\frac{F13-2.197}{0.986}$
			37H	0.966	6.11	$F11=\frac{F13-6.11}{0.966}$
F17 (and F18) to F13	W. Meier (NSIDC)	Arctic (Summer)	19H	0.979	1.646	$F13=\frac{F17-1.646}{0.979}$
			37H	0.999	0.649	$F13=\frac{F17-0.649}{0.999}$

Users of the prior version of the AHRA MO date data product (V4) should note that the sensor used for 2017 MO dates has changed from F17 to F18 (Table 1) which results in slight differences in MO dates for most of the Arctic in 2017. Specific differences due to the sensor change for 2017 are discussed further in the Technical Validation section.

### Sea ice concentrations

Passive microwave sea ice concentrations (SIC) are used in the AHRA methodology to create annual masks of the maximum Arctic sea ice extent (SIE) preceding the start of the melt season. SIC data for the Northern Hemisphere were obtained from the NOAA/NSIDC Climate Data Record (CDR) of Passive Microwave Sea Ice Concentration, version 4^39^ data set. The NOAA/NSIDC CDR is available in the same 25 km north polar stereographic grid format as the Tb data and provides daily SIC from 25 October 1978 through 31 December 2022. The NOAA/NSIDC CDR version 4 uses temporal and spatial interpolation to fill (1) gaps in the SICs caused by missing or bad Tb swaths, (2) the region around the North Pole where passive microwave Tbs are not obtained (known as the pole hole), and importantly, (3) gaps in observations from the SMMR sensor which collected data every second day. The daily SICs used in this work are from the “cdr_seaice_conc” data field within the NOAA/NSIDC CDR product that uses SICs from the NASA Team^40^ and Bootstrap^41^ SIC algorithms to create a combined best estimate of SIC. The daily SICs from 1979–2022 were used to create the annual SIE masks as described below.

### Sea ice extent mask and algorithm starting date

Annual SIE masks are used to determine the grid cells with sea ice coverage at the beginning of the melt season and to identify grid cells where the AHRA will work to find the MO date. In prior versions of the AHRA^17^, including AHRA V4^11^, the end of the sea ice growth season is assumed to occur at the beginning of March (DOY 61). A 50% SIC threshold was selected to omit regions with low SICs which could be erroneously flagged with a MO date due to the high concentration of open ocean within the grid cell, instead, focussing MO detection on the areas with more consolidated sea ice conditions. A prior version of the NOAA/NSIDC CDR used for AHRA V4 and earlier MO dates did not interpolate missing SICs from data outages or the missing days in SMMR years (Table 2). To avoid these gaps, the annual SIE masks for AHRA V4 were constructed by identifying all grid cells where the SIC was ≥50% on one or both of the first two days with valid SIC data in March, which, given the every second day observations of SMMR, could span the 1–4 March period^11^. However, the date of maximum SIE does not always occur in the first days of March (Table 4). Here, an improved method is used to identify the annual SIE maxima based on a 50% SIC threshold to create annual SIE masks and to determine the date of the SIE maxima for MO date detection that more accurately represents the inter-annual variability in Arctic sea ice coverage present at the beginning of the melt season.

**Table 4:** Summary of annual SIE maxima for AHRA V5 SIE masks and algorithm starting dates and the mean differences between AHRA V5 and V4 MO dates during overlap years.

Year	SIE 50% V5 Max. (× 10^4^ km^2^)	SIE 50% V5 Max. Date	SIE 50% V5 Max. DOY	V5 Algorithm Starting Date (DOY)	V5 - V4 Mean Diff. (d)
1979	16.10	1-Mar	60	61	−0.7
1980	15.71	5-Mar	65	66	1.3
1981	15.24	14-Mar	73	74	5.7
1982	15.85	3-Mar	62	63	0.4
1983	15.82	14-Mar	73	74	5.4
1984	15.31	18-Mar	78	79	6.8
1985	15.60	17-Mar	76	77	8.1
1986	15.78	10-Mar	69	70	3.8
1987	15.74	24-Feb	55	56	−1.5
1988	15.86	9-Mar	69	70	2.3
1989	15.22	28-Feb	59	60	−1.0
1990	15.68	10-Mar	69	70	3.3
1991	15.17	25-Feb	56	57	−0.7
1992	15.06	22-Feb	53	54	−2.0
1993	15.47	8-Mar	67	68	2.2
1994	15.24	3-Mar	62	63	−0.2
1995	14.85	24-Feb	55	56	−1.7
1996	14.92	21-Feb	52	53	−3.8
1997	15.14	15-Feb	46	47	−4.5
1998	15.52	24-Feb	55	56	−0.8
1999	15.06	26-Feb	57	58	−0.1
2000	14.90	3-Mar	63	64	0.1
2001	15.23	2-Mar	61	62	−0.3
2002	15.09	19-Feb	50	51	−3.2
2003	15.15	2-Mar	61	62	−0.2
2004	14.78	10-Mar	70	71	3.7
2005	14.40	6-Mar	65	66	1.4
2006	14.22	10-Mar	69	70	3.2
2007	14.26	10-Mar	69	70	1.9
2008	14.84	9-Mar	69	70	3.7
2009	14.70	2-Mar	61	62	−0.6
2010	14.80	31-Mar	90	91	8.5
2011	14.15	16-Mar	75	76	4.1
2012	14.77	16-Mar	76	77	5.7
2013	14.64	28-Feb	59	60	−0.9
2014	14.47	20-Mar	79	80	7.1
2015	14.03	22-Feb	53	54	−2.0
2016	13.96	11-Mar	71	72	3.3
2017	13.91	14-Mar	73	74	5.8
2018	13.93	15-Mar	74	75	—
2019	14.27	11-Mar	70	71	—
2020	14.54	4-Mar	64	65	—
2021	14.27	10-Mar	69	70	—
2022	14.26	22-Feb	53	54	—
Mean	14.95	—	64.9	65.9	1.6

**Fig. 1:**
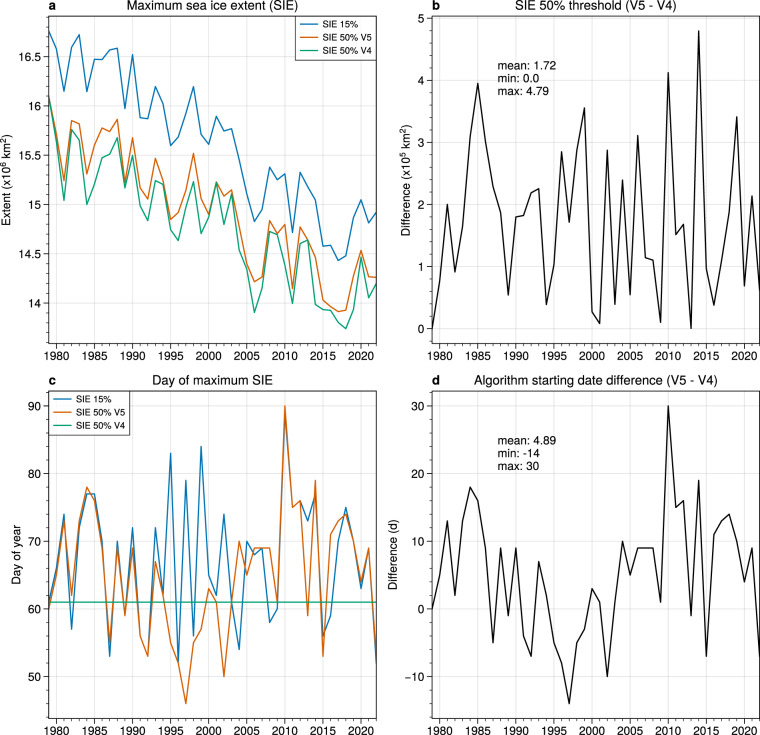
Comparison of Arctic SIE methods for AHRA sea ice masks. Panel (**a**) compares the annual SIE maxima for the traditional 15% SIC threshold (noted as SIE 15%), the annual SIE maxima based on a 50% SIC threshold used in AHRA V5 (noted as SIE 50% V5), and the SIE maxima based on a 50% SIC threshold on day of year 61 to approximate the SIE masks in AHRA V4 (noted as SIE 50% V4). Panel (**b**) shows the SIE difference between AHRA V5 and V4 SIE masks. Panel (**c**) compares the day of year of the above three SIE maxima and panel (**d**) shows the difference in algorithm starting dates between AHRA V5 and V4.

To inform the AHRA V5 methodology for creating SIE masks and determining the algorithm starting dates, an analysis of annual Arctic SIE maxima was completed. For general polar sea ice monitoring, daily SIE is computed as the areal sum of all grid cells where the SIC is ≥15% assuming that the pole hole region is ice covered^42,43^. The annual SIE maximum is the maximum daily SIE for the given year as shown in Fig. 1a (blue line, hereafter noted as SIE 15%). The DOY on which the annual SIE maxima occurs during 1979–2022 period varies from 52 (21 February) to 89 (30 March) with a mean of 67.1 and range of approximately 5.5 weeks (Fig. 1c, blue line).

Recognizing that similar inter-annual variability exists for SIEs computed with a 50% SIC threshold as used for the AHRA SIE masks, SICs from the NOAA/NSIDC CDR were used to compute the annual SIE 50% maxima as above (hereafter noted as SIE 50% V5). The annual SIE 50% V5 maxima are shown in Fig. 1a (orange line). Compared to the SIE 15% maxima (blue line), the SIE 50% V5 annual maxima (orange line) are approximately 1 × 10^6^ *km*^2^ smaller, due to the exclusion of lower SIC areas. To compare the SIE 50% V5 annual maxima to the fixed-date SIE used for AHRA V4, we approximate the AHRA V4 sea ice mask SIEs by computing the SIE 50% on DOY 61 (Fig. 1a (green line), hereafter noted as SIE 50% V4). The SIE 50% V4 maxima are generally lower than that of SIE 50% V5 (Fig. 1a), with a mean difference of 1.72 × 10^5^ *km*^2^ (Fig. 1b) indicating that the fixed date period for estimating the annual SIE 50% V4 maxima underestimates the SIE 50% V5 maxima in most years.

The DOY on which the three different SIE maxima occurred is shown in Fig. 1c. Note that the SIE 50% V5 maxima dates (orange line) are similar to the SIE 15% maxima dates (blue line) for much of the period with most larger deviations occurring in the 1995–2005 period. When compared to the approximated SIE 50% V4 maxima date (fixed on DOY 61), the inter-annual variablity of the SIE 50% V5 maxima date is quite high (Fig. 1c). If we assume that the melt season begins on the day following the date of the SIE 50% V5 maxima, there is a mean difference of nearly 5 d between the V5 and V4 AHRA algorithm starting dates, with a range of approximately 1.5 months (Fig. 1d). Thus, by taking into account the high inter-annual variability of the SIE 50% maxima, we can better represent the actual state of Arctic sea ice coverage present at the beginning of the annual melt season and incorporate inter-annual variability in timing of the onset of the summer melting season for AHRA V5 in line with other existing measures of the Arctic seasonal sea ice cycle^43^.

Based on the SIE analysis above, the AHRA V5 annual SIE masks used to identify the sea ice grid cells where the AHRA will identify a MO date are defined as the sea ice grid cells where the SIC is ≥50% on the date of the annual SIE 50% V5 maxima (Fig. 1, Table 4). For consistency with the gap in data coverage of Tb observations around the North Pole, the interpolated SICs within the pole hole region were removed from the annual SIE masks prior to computing MO dates. The algorithm starting date which corresponds to the first possible MO date for the year is defined as the day following the date of the SIE 50% V5 maxima (Table 4). Thus, AHRA V5 implements a new variable algorithm starting date methodology, that varies in response to inter-annual variablity in the annual SIE maxima, improving on problems related to the fixed algorithm starting date (i.e., DOY 61) identified in previous investigations^11,17^.

### The advanced horizontal range algorithm

The AHRA was developed by Drobot and Anderson^1^ to identify the MO date over first-year and perennial Arctic sea ice as an improvement to the original methodology of Anderson^44^. The AHRA method has since been updated periodically by Bliss and Anderson^11,17^. Microwave emission of snow and ice surfaces is proportional to the physical temperature and emission properties of the material^45^. When frozen snow and ice surfaces become wet from melting, they become a strong source of microwave emission approaching blackbody values. The AHRA utilizes this property by computing the horizontal range (HR) of Tbs from the 19H and 37H channels expressed as *HR* = *Tb*_19*H*_−*Tb*_37*H*_. The 37H channel is affected more strongly by melt than the 19H channel^3,44^, causing the time series of daily HR values to shift toward 0 or negative values when melting begins^1^.

**Fig. 2:**
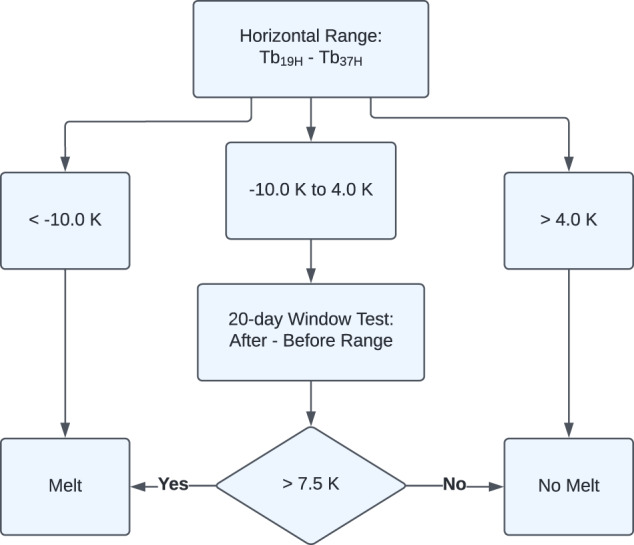
Schematic of the Advanced Horizontal Range Algorithm. Adapted from Drobot and Anderson^1^.

To derive MO dates for a year, the AHRA computes the daily HR using the 19H (18H for SMMR) and 37H channels for each sea ice grid cell identified in the year’s SIE mask^1^ (Fig. 2). The AHRA then steps through the time series of the daily HR beginning with the first day of the melt season as defined by the algorithm starting date methodology above (Table 4). If the HR for the day is <−10.0 K, melt conditions are assumed and the algorithm assigns the grid cell with the DOY for MO. If the HR is >4.0 K, freezing, winter conditions are assumed and the algorithm proceeds to the next day. If the HR is within the −10.0 K to 4.0 K range (inclusive) the melt status is ambiguous and a test is performed over a 20-day window. The 20-day window test checks the 10 days prior to and 9 days following the current day for a sustained step change in the HR time series consistent with the onset of the melt season. The range of the HR time series (i.e., *HR*_*max*_−*HR*_*min*_) in the 10 days prior is computed and compared to the range of the HR time series from the 9 days after. If the difference between the “after” range and the “before” range is >7.5 K, then melt is assumed and the algorithm assigns the MO date. If the “after” and “before” range is <7.5 K, then winter conditions are assumed to persist and the algorithm proceeds to the next day.

The result of the AHRA is a gridded array for the year containing the DOY on which the algorithm identified MO for sea ice covered areas. The AHRA with updated SIE masking and variable starting date methodology was used to produce the annual arrays of Arctic MO dates for 1979–2022 contained in the AHRA V5 data set.

### Computation of additional data set variables

The AHRA V5 data set contains additional gridded data fields that provide basic summary statistics of the MO dates computed over the full 1979–2022 data set. The summary statistics computed include the mean, median, standard deviation, earliest MO date, latest MO date, range of MO dates (expressed as latest – earliest), and the linear trend of MO dates (expressed as *ddec*^−1^) for grid cells at which a MO date was found in all 44 years of the time series. The above statistical summary variables are included as a component of the AHRA V5 data product.

## Data Records

**Fig. 3:**
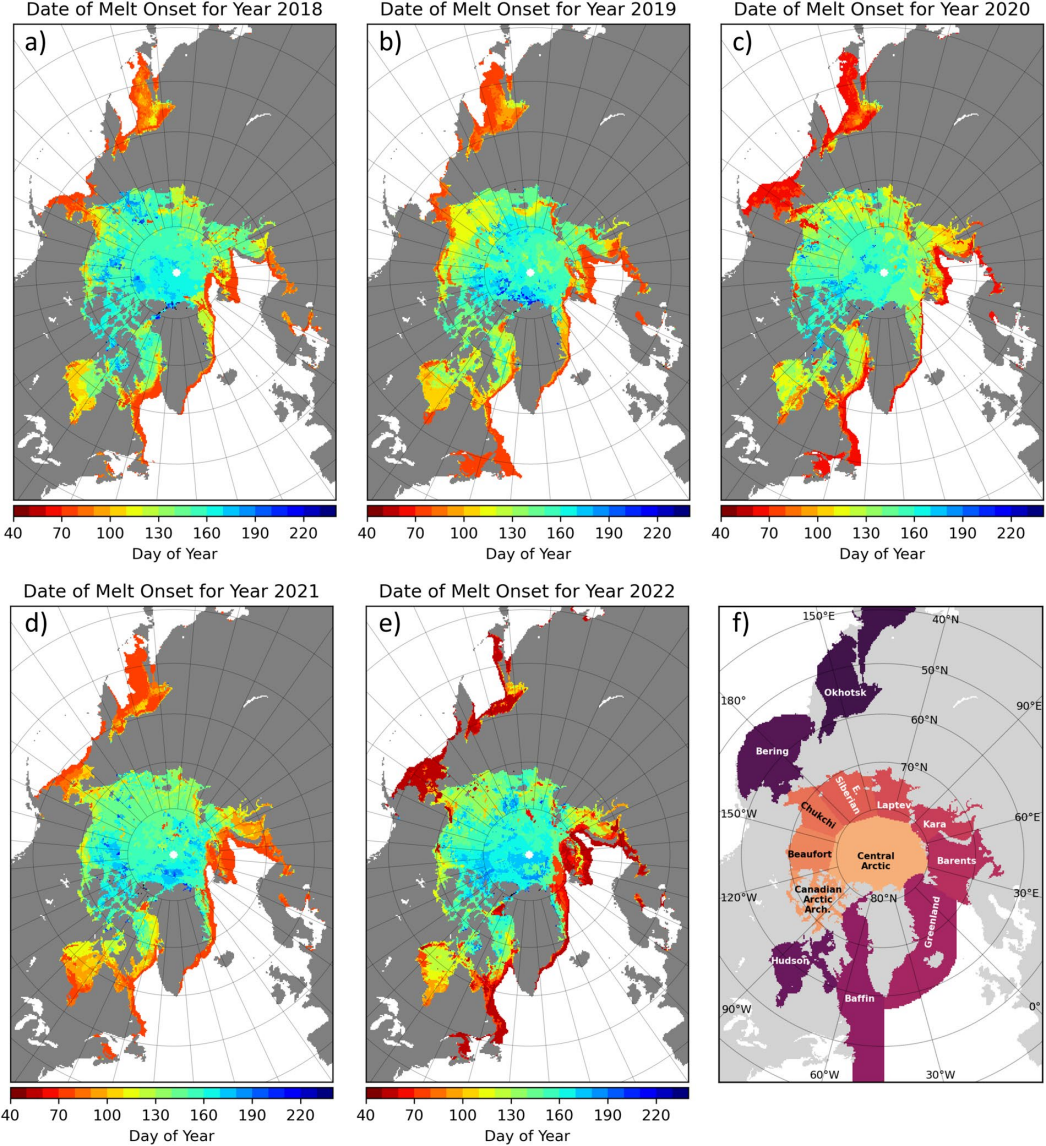
In panels (**a**–**e**), sample images of annual maps of recent Arctic sea ice melt onset (MO) dates for 2018–2022 distributed with the data set. Dates are expressed as the day of year (DOY) from the first of the year. In panel (**f**), a map of Arctic sub-regions.

**Fig. 4:**
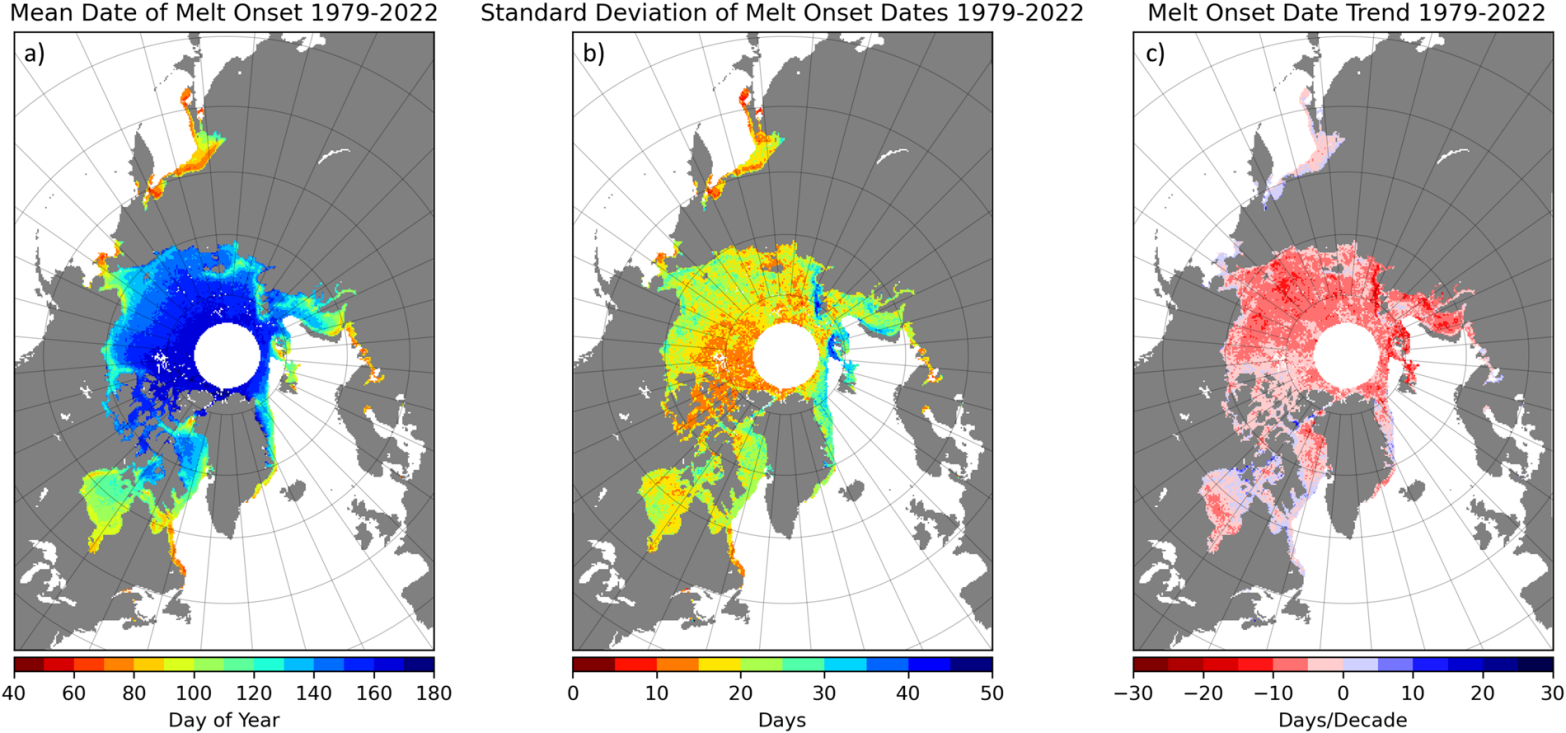
Sample images of Arctic sea ice melt onset (MO) date summary statistics from 1979–2022 provided in the data set. Maps show (**a**) the mean, (**b**) standard deviation and (**c**) decadal trend of MO dates for grid cells with a valid MO date in all 44 years. Areas in white denote grid cells where a MO date did not occur in one or more years, the pole hole data gap, and ice-free ocean.

The AHRA V5 MO date data set for years 1979–2022 presented in this paper is available for download at Zenodo^32^. The data are also available at the NASA DAAC repository at NSIDC^31^; however, the archived data set at NSIDC will be periodically updated with new annual MO dates in the future. The AHRA V5 data set consists of a single self-describing Network Common Data Form 4 (NetCDF-4) file containing gridded arrays of the annual MO dates for each year (1979–2022) and gridded arrays providing the 1979–2022 summary statistics of the data set. The MO date arrays are stored as a variable named snow melt onset date (SMOD) with a time dimension length of 44. The netCDF-4 file also contains variables giving the latitude and longitude positions for each grid cell as well as the north polar stereographic grid projection information. In addition to the primary data file, a collection of image files in Portable Network Graphics (.png) format are provided that show quick-look maps of each of the data layers contained in the netCDF-4 file. A sample of annual MO date images for the new years added to the AHRA V5 data set (2018–2022) are shown in Fig. 3a–e and a sample of images for some of the 1979–2022 summary statistics fields are shown in Fig. 4.

Data within the 44 gridded MO date arrays are stored as integer values giving the DOY of MO at each sea ice grid cell or a flag value indicating why the cell does not contain a MO date (Table 5). The full range of integers used for valid MO dates is 40 to 245 (Table 5) while the actual lowest MO date value for any given year is the corresponding algorithm starting date (Table 4, Fig. 1c). Negative data values as indicated in Table 5 are used to flag grid cells where open water including lakes and ice-free ocean, the pole hole data gap, and land cells are located. In a few cases, a MO date was not detected during the year’s melt season, primarily in early years of the time series. The grid cells where a MO date was not found were assigned a value of 255. For the statistical summary data layers in the product, data are stored as floating point values. Negative data values are used to flag grid cells with no data because the statistic was not computed, the pole hole region above 84.5°N, and land cells as listed in Table 6. The valid range of data values for the computed statistics ranges from −130 to 255.

**Table 5:** Description of data values used in annual MO date gridded arrays.

Value	Description
−150	Open water (ocean and lakes)
−100	Pole hole; no data available
−50	Land mask
40 to 245	MO date, expressed as day of year (DOY)
255	No MO date identified

**Table 6:** Description of data values used in the MO date summary statistics gridded arrays.

Value	Description
−150	No data
−100	Pole hole; no data available
−50	Land mask
−130 to 255	Valid data

## Technical Validation

To assess the quality of the new MO date data product and quantify the impact of V5 updates on the Arctic MO date data record, mean Arctic-wide differences between AHRA V5 and AHRA V4 MO dates are examined for the overlap period 1979–2017. Three example years are then used to illustrate the primary types of regional differences users should expect when using the updated V5 data set. Finally, the AHRA V5 MO dates are compared to another independently-produced passive microwave MO data product for the Arctic region and commonly used Arctic sub-regions (Fig. 3f).

### Differences between AHRA V5 and V4 MO dates, 1979–2017

#### Arctic-wide differences

To compare the impact of the new methodology introduced in AHRA V5 on the overall record of annual Arctic MO dates, the mean differences between AHRA V5 and V4 MO dates were computed for each year from 1979–2017. The differences in d (expressed as *V*5*V*4) were computed from the mean of all grid cells with a valid MO date in each year (Table 4). The mean differences are not systematically negative or positive, as a result of the variability in AHRA V5 algorithm starting dates prior to and after the fixed algorithm starting date of V4 (DOY 61). The mean differences range from |0.1| d in 1999 and 2000 to |8.5| d in 2010 with an overall mean difference of 1.6 d (Table 4). Thus, on average, the changes implemented by the V5 update delay the mean Arctic-wide MO date by 1.6 d over the full overlap period with mean differences no larger than 8.5 d in any individual year. Next we consider how MO dates are impacted by differences in AHRA V5 processing at the regional scale.

#### Regional differences

**Fig. 5:**
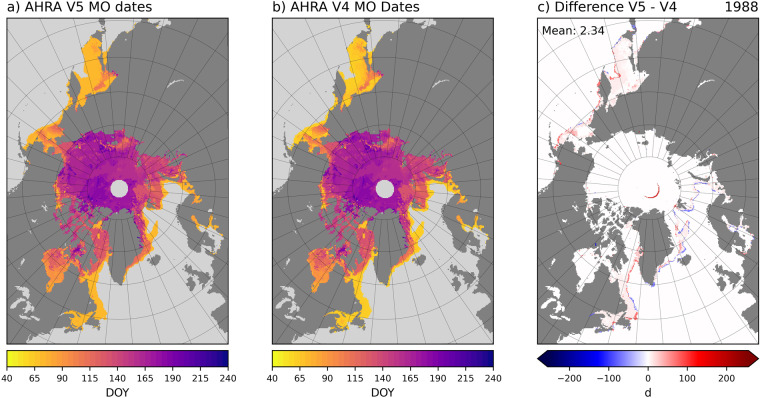
Comparison between AHRA V5 and V4 MO dates for 1988. Differences are expressed as V5 – V4. The arc of positive differences in the central Arctic in panel (**c**) is an artifact of slight differences between pole hole masks.

For most years, differences between AHRA V5 and V4 MO dates are primarily confined to areas near the sea ice edge and in the marginal ice zone. Figure 5 shows the differences in 1988 as an example of a typical MO year. Larger differences in Fig. 5c are found along the perimeter of the SIE, while areas of smaller differences are found generally poleward of the sea ice edge in the more consolidated sea ice areas of regions such as the Sea of Okhotsk, Bering and Greenland Seas, and in Baffin Bay (see Fig. 3f for region names). The larger difference areas along the sea ice edge are generally a result of slight differences in the extent of the SIE masks between V5 and V4 where positive differences (red) show sea ice locations in V5 that do not exist in V4 and negative differences (blue) show the reverse. The smaller differences that appear away from the immediate ice edge, for example, within the Sea of Okhotsk, Bering and Greenland Seas, and Baffin Bay regions are primarily a result of differences in the algorithm starting date and, thus, the earliest possible date of MO. In 1988, the algorithm starting date for V5 is DOY 70, 9 days later than the arbitrary DOY 61 algorithm starting date of V4 (Table 4) and primarily positive MO date differences are found. Therefore, the earliest MO dates in V5 are later than those from V4 which were obtained when the SIE was still expanding. Additional differences near the pole hole appear as an arc shape in 1988 (Fig. 5c) and in the other SSM/I years from 1989–2007 (not shown). Differences in this region are an artifact of slight differences in the pole hole mask used in AHRA V4 for SSM/I data (>87.2 °N)^30^ and the pole hole as defined in the NOAA/NSIDC CDR SIC data used for AHRA V5 sea ice masks^39^. This artifact results in a small number of grid cells around the pole hole where MO dates are now computed in AHRA V5 that were not previously available in AHRA V4. For 1988 the mean difference in MO dates for all sea ice grid cells where at least one version produced a MO date is 2.34 days (Fig. 5c).

**Fig. 6:**
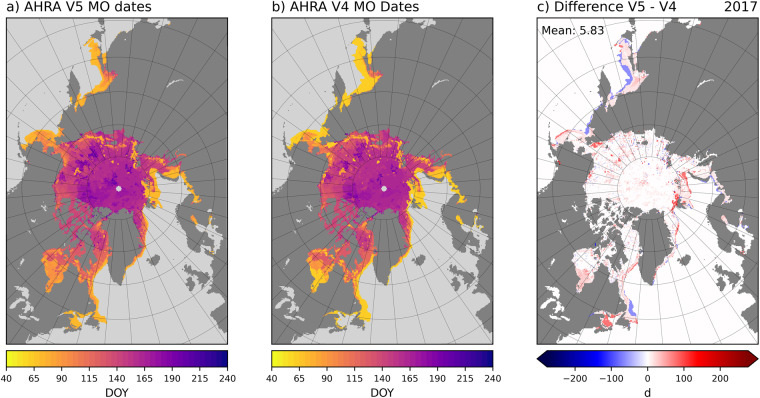
Comparison between AHRA V5 and V4 MO dates for 2017. Differences are expressed as V5 – V4. In 2017 the sensor switched from F17 (V4) to F18 (V5) producing small differences throughout the sea ice pack in the Arctic Ocean in addition to the expected differences at the sea ice edge.

For 2017, the larger differences between AHRA V5 and V4 MO dates are concentrated along the sea ice edge (Fig. 6c). Similar to 1988 described above (Fig. 5c), the ice edge differences are attributed to the SIE mask and algorithm starting date changes in AHRA V5. In contrast to 1988 and other years (not shown), 2017 also has grid cells with smaller differences scattered throughout most of the sea ice area including higher latitudes of the Arctic Ocean (Fig. 6c). Recall that the Tbs used to produce AHRA V4 MO dates for 2017 were from the of the SSMIS sensor on the F17 platform (Tables 1, 2). In May of 2016, the F17 SSMIS developed problems affecting the 37 GHz channel used to derive MO dates^38^. To avoid any impacts from poor Tb data quality in AHRA V5, the Tb data source was changed to the F18 platform when these data first become available in the DMSP SSM/I-SSMIS Daily Polar Gridded Brightness Temperatures, version 6^25^ data set on 1 January 2017 (Table 2). The scattered differences throughout the sea ice pack seen in 2017 (Fig. 6c) are primarily due to the sensor change from F17 to F18. Although 2017 is the only year in the AHRA V5 time series affected by a sensor change producing a larger number of grid cells with a MO date difference, the mean difference between AHRA V5 and V4 is only 5.83 d (Fig. 6c), well below the 8.5 d maximum mean difference found during the overlap years (Table 4).

**Fig. 7:**
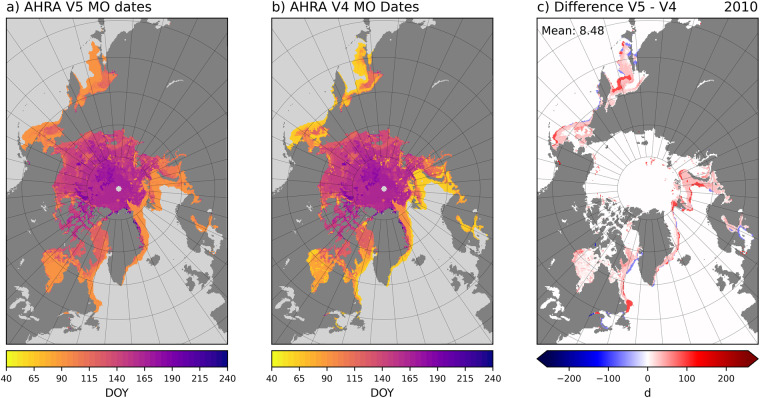
Comparison between AHRA V5 and V4 MO dates for 2010. Differences are expressed as V5 – V4.

2010 is an example of an extreme difference year where the mean difference between AHRA V5 and V4 is 8.48 d (Fig. 7), the largest mean MO date difference of the 1979–2017 overlap years (Table 4). In this case, the majority of *V*5*V*4 differences are positive (red) and are generally confined to the peripheral sea ice regions including the Sea of Okhotsk; Bering, Barents, and Greenland Seas; and the southern portions of Baffin Bay (Fig. 7). The annual SIE 50% V5 maximum was unusually late this year, occurring on DOY 90 (31 March, Table 4) one month later than the V4 algorithm starting date on DOY 61, and interestingly, the same date as the traditional SIE 15% minimum (Fig. 1c). The largest positive differences along the sea ice edge (red) in 2010 represent sea ice locations that continued to grow in the month following the assumed annual SIE maximum in AHRA V4 around DOY 61 (Fig. 7). As a result, the MO dates in these peripheral sea ice regions are generally much later than in V4 since the algorithm starting date and the first possible MO date is DOY 91. It should be noted that the methodology changes for AHRA V5 primarily affect MO dates in the peripheral sea ice regions at lower latitudes than in the central Arctic at higher latitudes in all of the overlap years. Thus, for most of the Arctic Ocean only small differences in the MO dates are found at a few scattered grid cells as in 2010 (Fig. 7c) and in most other years of the data record excluding 2017 as discussed above (Fig. 6c).

### Comparison between AHRA V5 and passive microwave MO dates, 1979–2022

**Fig. 8:**
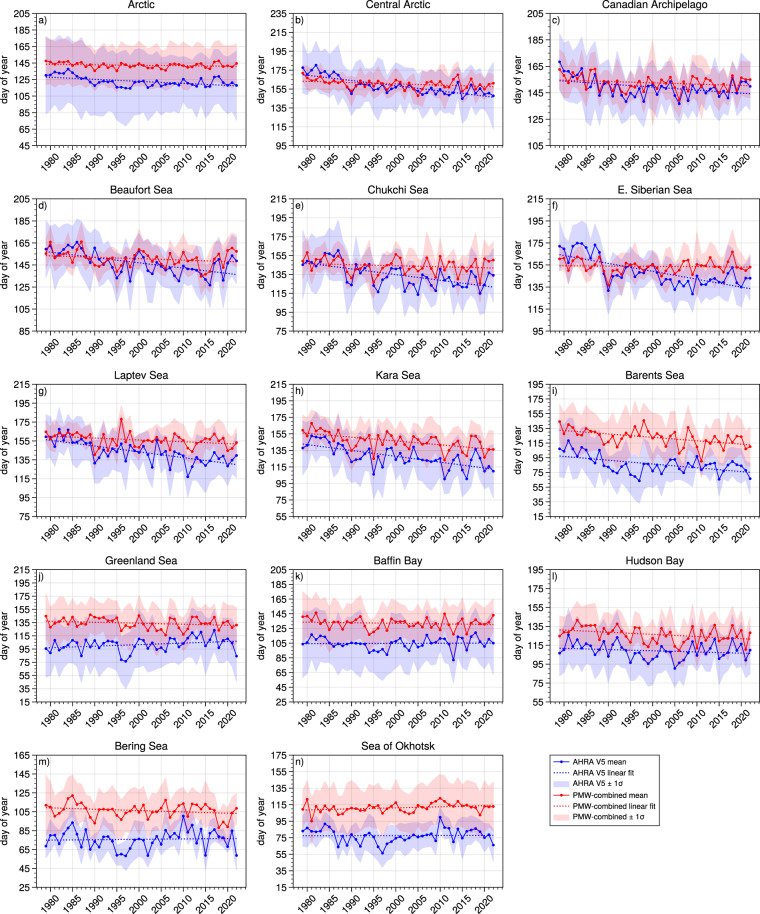
Comparison between annual mean MO dates from AHRA V5 (blue) and PMW-combined (red) data for the Arctic and Arctic sub-regions as shown in Fig. 3f. Shaded regions denote ±1 *σ* around the mean. Dotted lines are from a least squares linear regression.

To further assess the quality of AHRA V5 data, MO dates for the 1979–2022 period are compared to an independent set of passive microwave-derived Arctic sea ice melt and freeze onset dates developed by Markus *et al*.^3^. The Markus *et al*. method^3^ (hereafter called the Passive Microwave or PMW algorithm) data product contains annual gridded arrays of the early and continuous MO dates for Arctic sea ice as well as the dates of early and continuous freeze-up in the autumn and winter. The PMW MO dates are provided in the 25 km NSIDC north polar stereographic grid and data for 1979–2022 were obtained from the NASA Cryospheric Sciences Data website (https://earth.gsfc.nasa.gov/cryo/data/arctic-sea-ice-melt; product updated 20 April 2023, last accessed 1 August 2023). As noted previously, the AHRA detects the first day of the year that melting occurs which is most comparable to the PMW early MO date. However, the PMW algorithm does not detect an early MO date in cases when the sea ice and snow surfaces do not re-freeze after initial melting, even for short periods, until seasonal freeze-up begins the following autumn. In this case, only a continuous MO date is provided in the PMW data set. Therefore, to directly compare AHRA V5 to PMW MO dates, the PMW early and continuous MO dates are combined such that the grid cells where an early MO date was not found are filled with the continuous MO date following Bliss *et al*.^29^. That is, the earliest date of the early and continuous MO dates for the year (hereafter called the PMW-combined MO date) are used for comparison with AHRA V5. The mean and standard deviation of MO dates for the Arctic and sub-regions (Fig. 3f) was computed from all grid cell locations with a valid MO date for each year. The time series of mean MO dates from the AHRA V5 and PMW-combined data sets are shown in Fig. 8 with shading indicating ±1 standard deviation (*σ*) around the mean. The average of the annual means and standard deviations for each region over the 44 year time series was then computed and is reported in Table 7.

**Table 7:** Regional mean and standard deviation of MO dates from the AHRA V5 and PMW-combined data sets, 1979–2022. The mean difference is expressed as AHRA V5 − PMW-Combined. Regions correspond to locations as defined in Fig. 3f.

Region	AHRA V5 Mean	AHRA V5 Std. Dev.	PMW-combined Mean	PMW-combined Std. Dev.	Mean MO Date Difference
Arctic	122.7	5.9	142.3	3.1	−19.6
Central Arctic	158.2	8.9	160.4	4.9	−2.2
Canadian Arctic Archipelago	149.2	7.1	152.5	5.4	−3.3
Beaufort	146.7	10.0	150.6	7.0	−3.9
Chukchi	134.5	12.4	144.2	7.9	−9.6
E. Siberian	148.9	12.7	153.7	5.5	−4.8
Laptev	143.2	11.1	156.0	7.2	−12.8
Kara	127.5	13.1	146.2	10.2	−18.7
Barents	86.2	12.3	122.5	11.3	−36.3
Greenland	101.8	10.4	134.0	7.9	−32.2
Baffin	104.7	7.7	132.0	7.3	−27.3
Hudson	109.1	8.1	126.4	8.1	−17.3
Bering	75.9	9.9	106.3	7.3	−30.4
Okhotsk	77.6	8.3	111.3	5.4	−33.7

For the Arctic as a whole (Fig. 8a), AHRA V5 MO dates are consistently earlier in the year than the PMW-combined with both time series falling within ±1 *σ* of the other. The overall mean MO date for all Arctic sea ice over the 1979–2022 data record from AHRA V5 is DOY 122.7 (2 May) and DOY 142.3 (22 May) for PMW-combined (Table 7). Similar to a previous comparison by Bliss *et al*.^29^, the early bias of MO dates from the AHRA when compared to PMW MO dates is expected due to differences in the algorithm methodology including the algorithm starting dates. In particular, the AHRA V5 algorithm starting dates vary from year to year with the earliest possible MO dates in the data record occurring on DOY 51 (Table 4). The earliest possible MO date for PMW-combined is DOY 75^3^, more than 3 weeks later than the earliest MO dates in AHRA V5 contributing to some of the late bias of the PMW-combined MO (Fig. 8a). Further, the PMW algorithm detects a MO date by computing three different melt-indicating parameters from Tbs and using a weighting function to identify the most likely MO date based on the combination of parameters^3^. An additional spatial filter is then applied to reduce the effects of spurious Tbs by testing neighboring grid cells for spatial coherency before assigning the MO date. The PMW algorithm methodology is, therefore, a more conservative strategy for detecting MO dates than that utilized by the AHRA^29^ which also contributes to the observed delay in PMW-combined MO dates relative to AHRA V5.

Dividing the Arctic into sub-regions (Fig. 8b–n) shows regional variability in the magnitude of differences between AHRA V5 and PMW-combined MO dates. Most regions at the northern-most latitudes and within the Arctic Ocean including the Central Arctic, Canadian Arctic Archipelago, and the Beaufort, Chukchi, East Siberian, Laptev, and Kara Seas and also the Hudson Bay region show differences that are generally smaller than the full Arctic (Fig. 8). The overall mean differences for the above sub-regions varies from |2.2| d in the Central Arctic to |18.7| d in the Kara Sea (Table 7). Greater regional differences are present for the regions at lower latitudes and along the periphery of the sea ice pack ranging from |27.3| d in Baffin Bay to |36.3| d in the Barents Sea region (Table 7, Fig. 8). The regional differences in MO dates demonstrate similar performance between the two algorithms at locations where SICs are generally high and uncertainties in passive microwave observations are low including most areas of the Arctic Ocean where mean differences are typically <|10| d, regionally as low as |2.2| d (Table 7). Larger differences are primarily found in regions along the sea ice periphery where the AHRA and PMW algorithms diverge with respect to the methods for defining the algorithm starting dates and uncertainties in sea ice products are elevated due to lower SICs and the presence of more mobile, less compacted sea ice (e.g., the marginal ice zone). Results of the above regional comparison between AHRA V5 and PMW-combined MO dates increases confidence that the AHRA V5 MO dates are producing reasonable estimates of the annual MO date timing in the high SIC regions of the Arctic Ocean with higher uncertainty in peripheral ice regions.

## Usage Notes

There are several limitations affecting estimates of MO timing common to all passive microwave MO date algorithms including the AHRA. Passive microwave satellite observations are often used to monitor sea ice conditions in the polar regions because microwave emission from the surface can be measured without the need for sunlight and in cloudy sky conditions which are common in the Arctic summer. Cloud cover is largely transparent to radiation at microwave frequencies used to derive MO dates except in cases where liquid water clouds contaminate the signal^46^. The AHRA methodology assumes that liquid water clouds from passing cyclones are short-lived and that contamination from these clouds would not last beyond synoptic timescales^1^. Thus, the 20-day window (Fig. 2) is used to filter instances of short-lived atmospheric contamination by testing the 20 day period surrounding a potential MO date for a step change in the daily HR time series from winter-like HR values to summer-like HR values that signals the onset of the summer melting season. A previous study by Bliss *et al*.^29^ compared MO dates from the AHRA with the PMW algorithm data product and found that the primary limitation present in these data sets are inaccurate MO date estimates in the peripheral regions of the Arctic, near the sea ice edge where SICs are generally low which can be mistaken as seasonal surface melting. AHRA V4 and earlier fixed the algorithm starting date around an assumed SIE maximum date (DOY 61) which is shown in this work to vary from year to year over a 5.5 week range (Table 4). In AHRA V4 the earliest MO dates of a given year near the sea ice edge cluster at DOY 61 as an artifact of the fixed algorithm starting date^29^ (e.g., Figs. 5–7b). As shown in this work, the SIE maxima date is not always near DOY 61 (Fig. 1c, Table 4) which causes the AHRA to try detecting sea ice MO dates at grid cell locations where the SIE is still growing. Thus, inaccurate MO dates can be produced where the SIE has not yet been established for the year in the peripheral sea ice regions. The updated SIE mask and variable algorithm starting dates used in AHRA V5 are implemented to improve this limitation present in prior passive microwave MO date data sets; however, local variation in the SIE maxima can still cause uncertainty in the MO dates for peripheral sea ice regions. Users of AHRA V5 and other passive microwave MO date products should still use caution when using MO dates near the sea ice edge.

In previous work, passive microwave MO dates from the AHRA V4 were compared with MO dates estimated from 2 m surface air temperatures (SAT) from satellite and interpolated buoy observations finding overall biases approximately 11 days with the passive microwave MO occurring earlier than the SAT-estimated MO^11^. This difference between SAT and passive microwave MO dates is expected since two different surfaces are being measured. The microwave emission is from the ice or snow surface to a depth of a few cm in snow pack^45^, while the SAT is measured above the surface and can differ from the snow or ice surface temperature^47^. Melting in the snow modifies the snow grains resulting in increased grain size and more volume scattering, increasing variability in microwave emission consistent with MO^3^. This change in microwave emission from melting snow occurs even due to very short excursions above the melting point during peak diurnal temperatures which may not appear in daily averaged SAT measurements^11^. Users should, therefore, expect MO dates from passive microwave observations such as AHRA V5 to precede MO date estimates derived from SATs.

Finally, the passive microwave MO date observations are obtained from daily averaged Tbs gridded at a nominal spatial resolution of 25 km. Due to the coarse spatial resolution of the passive microwave data, users should use caution when comparing passive microwave MO dates with observations from ground-based temperature observations such as from buoys or other *in situ* instruments. These observations are often point observations falling within a much larger area represented by a satellite grid cell (25 km × 25 km). Passive microwave MO date methodologies, including the AHRA, were initially validated against buoy or other *in situ* observations of SATs^1,3^, but the MO date at a single grid cell may not correspond directly to other indicators of surface melting observed from a point location and direct comparisons should be avoided.

## Data Availability

The code used to produce the AHRA V5 MO dates is archived at Zenodo^48^. Additional code used to complete the analysis and produce figures in this paper is available at Zenodo^49^.

## References

[1] Drobot SD, Anderson MR (2001). An improved method for determining snowmelt onset dates over Arctic sea ice using Scanning Multichannel Microwave Radiometer and Special Sensor Microwave/Imager data. Journal of Geophysical Research: Atmospheres.

[2] Livingstone CE, Singh KP, Gray AL, Member S, Gray AL (1987). Seasonal and regional variations of active/passive microwave signatures of sea ice. IEEE Transactions on Geoscience and Remote Sensing.

[3] Markus, T., Stroeve, J. C. & Miller, J. Recent changes in Arctic sea ice melt onset, freezeup, and melt season length. *Journal of Geophysical Research: Oceans* **114**, 10.1029/2009JC005436 (2009).

[4] Peng, G., Steele, M., Bliss, A. C., Meier, W. N. & Dickinson, S. Temporal means and variability of Arctic sea ice melt and freeze season climate indicators using a satellite climate data record. *Remote Sensing* **10**, 10.3390/rs10091328 (2018).

[5] King MD, Veron DE, Huntley HS (2020). Early predictors of seasonal Arctic sea-ice volume loss: the impact of spring and early-summer cloud radiative conditions. Annals of Glaciology.

[6] Maksimovich E, Vihma T (2012). The effect of surface heat fluxes on interannual variability in the spring onset of snow melt in the central Arctic Ocean. Journal of Geophysical Research: Oceans.

[7] Bliss AC, Anderson MR (2014). Daily area of snow melt onset on Arctic sea ice from passive microwave satellite observations 1979–2012. Remote Sensing.

[8] Liu Z, Schweiger A (2017). Synoptic conditions, clouds, and sea ice melt onset in the Beaufort and Chukchi seasonal ice zone. Journal of Climate.

[9] Mortin J (2016). Melt onset over Arctic sea ice controlled by atmospheric moisture transport. Geophysical Research Letters.

[10] Hegyi BM, Deng Y (2017). Dynamical and thermodynamical impacts of high- and low-frequency atmospheric eddies on the initial melt of Arctic sea ice. Journal of Climate.

[11] Bliss AC, Anderson MR (2018). Arctic sea ice melt onset timing from passive microwave-based and surface air temperature-based methods. Journal of Geophysical Research: Atmospheres.

[12] Perovich DK, Nghiem SV, Markus T, Schweiger A (2007). Seasonal evolution and interannual variability of the local solar energy absorbed by the Arctic sea ice-ocean system. Journal of Geophysical Research: Oceans.

[13] Steele M, Dickinson S (2016). The phenology of Arctic Ocean surface warming. Journal of Geophysical Research: Oceans.

[14] Winebrenner DP, Nelson ED, Colony R, West RD (1994). Observation of melt onset on multiyear Arctic sea ice using the ERS 1 synthetic aperture radar. Journal of Geophysical Research.

[15] Howell, S. E. L., Tivy, A., Yackel, J. J., Else, B. G. T. & Duguay, C. R. Changing sea ice melt parameters in the Canadian Arctic Archipelago: Implications for the future presence of multiyear ice. *Journal of Geophysical Research: Oceans* **113**, 10.1029/2008JC004730 (2008).

[16] Mahmud MS, Howell SEL, Geldsetzer T, Yackel J (2016). Detection of melt onset over the northern Canadian Arctic Archipelago sea ice from RADARSAT, 1997–2014. Remote Sensing of Environment.

[17] Bliss AC, Anderson MR (2014). Arctic sea ice melt onset from passive microwave satellite data: 1979–2012. The Cryosphere.

[18] Stroeve J, Markus T, Meier WN, Miller J (2006). Recent changes in the Arctic melt season. Annals of Glaciology.

[19] Wang L (2011). Integrated pan-Arctic melt onset detection from satellite active and passive microwave measurements, 2000–2009. Journal of Geophysical Research Atmospheres.

[20] Marshall, S., Scott, K. A. & Scharien, R. K. Passive microwave melt onset retrieval based on a variable threshold: Assessment in the Canadian Arctic Archipelago. *Remote Sensing* **11**, 10.3390/rs11111304 (2019).

[21] Rigor, I. G., Colony, R. L. & Martin, S. Variations in surface air temperature observations in the Arctic. *Journal of Climate* **13**, 896–914, 10.1175/1520-0442(2000)013<0896:VISATO>2.0.CO;2 (2000).

[22] Lindsay, R. W. Temporal variability of the energy balance of thick Arctic pack ice. *Journal of Climate* **11**, 313–333, 10.1175/1520-0442(1998)011<0313:TVOTEB>2.0.CO;2 (1998).

[23] Andreas EL, Ackley SF (1982). On the differences in ablation seasons of Arctic and Antarctic sea ice. Journal of the Atmospheric Sciences.

[24] Bliss AC, Steele M, Peng G, Meier WN, Dickinson S (2019). Regional variability of Arctic sea ice seasonal change climate indicators from a passive microwave climate data record. Environmental Research Letters.

[25] Meier WN, Stewart JS, Wilcox H, Scott DJ, Hardman MA (2021). NASA National Snow and Ice Data Center Distributed Active Archive Center.

[26] Peng G, Meier WN, Scott DJ, Savoie MH (2013). A long-term and reproducible passive microwave sea ice concentration data record for climate studies and monitoring. Earth System Science Data.

[27] Smith A, Jahn A, Wang M (2020). Seasonal transition dates can reveal biases in Arctic sea ice simulations. The Cryosphere.

[28] Smith, A. & Jahn, A. Definition differences and internal variability affect the simulated Arctic sea ice melt season. *Cryosphere* **13**, 10.5194/tc-13-1-2019 (2019).

[29] Bliss, A. C., Miller, J. A. & Meier, W. N. Comparison of passive microwave-derived early melt onset records on Arctic sea ice. *Remote Sensing* **9**, 10.3390/rs9030199 (2017).

[30] Anderson M, Bliss AC, Drobot S (2019). NASA National Snow and Ice Data Center Distributed Active Archive Center.

[31] Bliss AC, Anderson MR, Drobot S (2022). NASA National Snow and Ice Data Center Distributed Active Archive Center.

[32] Bliss AC (2023). Zenodo.

[33] Anderson M, Bliss AC, Drobot S (2014). NASA National Snow and Ice Data Center Distributed Active Archive Center.

[34] Drobot S, Anderson M (2001). NASA National Snow and Ice Data Center Distributed Active Archive Center.

[35] Drobot S, Anderson M (2009). NASA National Snow and Ice Data Center Distributed Active Archive Center.

[36] Gloersen P (2006). NASA National Snow and Ice Data Center Distributed Active Archive Center.

[37] Abdalati W, Steffen K, Otto C, Jezek K (1995). Comparison of brightness temperatures from SSMI instruments on the DMSP F8 and F11 satellites for Antarctica and the Greenland ice sheet. International Journal of Remote Sensing.

[38] Stewart, S., Wilcox, H., Meier, W. & Scott, D. Comparison of F17 and F18 daily polar gridded SSMIS data. *National Snow and Ice Data Center* https://nsidc.org/data/documentation/comparison-f17-and-f18-daily-polar-gridded-ssmis-data (2019).

[39] Meier WN, Fetterer F, Windnagel AK, Stewart JS (2021). National Snow and Ice Data Center.

[40] Cavalieri DJ, Gloersen P, Campbell WJ (1984). Determination of sea ice parameters with the NIMBUS 7 SMMR. Journal of Geophysical Research: Atmospheres.

[41] Comiso, J. C. Characteristics of Arctic winter sea ice from satellite multispectral microwave observations. *Journal of Geophysical Research* **91**, 10.1029/JC091iC01p00975 (1986).

[42] Parkinson CL, Cavalieri DJ, Gloersen P, Zwally HJ, Comiso JC (1999). Arctic sea ice extents, areas, and trend, 1978–1996. Journal of Geophysical Research: Oceans.

[43] Parkinson CL (2022). Arctic sea ice coverage from 43 years of satellite passive-microwave observations. Frontiers in Remote Sensing.

[44] Anderson MR (1987). Snow melt on sea ice surfaces as determined from passive microwave satellite data. Large Scale Effects of Seasonal Snow Cover: Proceedings of the Vancouver Symposium, August 1987.

[45] Ulaby, F. T., Moore, R. K. & Fung, A. K. *Microwave remote sensing: active and passive*, vol. 1–3 (Addison-Wesley Pub. Co., Reading, Mass, 1981).

[46] Cavalieri DJ, St Germain KM, Swift CT (1995). Reduction of weather effects in the calculation of sea-ice concentration with the DMSP SSM/I. Journal of Glaciology.

[47] Hall DK, Key JR, Casey KA, Riggs GA, Cavalieri DJ (2004). Sea ice surface temperature product from MODIS. IEEE Transactions on Geoscience and Remote Sensing.

[48] Bliss AC (2023). Zenodo.

[49] Bliss AC (2023). Zenodo.

[50] Jezek K (1991). BPRC Technical Report Number 91-03.

[51] Stroeve J, Maslanik J, Xiaoming L (1998). An intercomparison of DMSP F11- and F13-derived sea ice products. Remote Sensing of Environment.

